# Supplementary material for: Effect of Seminal Plasma Protein Fractions on Stallion Sperm Cryopreservation
Source: Int J Mol Sci. 2020 Sep 3;21(17):6415. doi: 10.3390/ijms21176415 (PMC7504567; doi:10.3390/ijms21176415)
Supplement: Supplementary file 1 [file ijms-21-06415-s001.pdf]

Table S1. Effect of SP protein fractions in three concentrations on sperm motility and kinematics parameters.

**Protein fraction (µg/mL)**	**TMOT (%)**	**PMOT (%)**	**ALH (µm)**	**BCF (Hz)**	**LIN (%)**	**STR (%)**	**VAP (µm/s)**	**VCL (µm/s)**	**VSL (µm/s)**	**WOB (%)**
**H^- 125**	18.8 ± 3.2	17.2 ± 3.5	4.5 ± 0.06 [1,b]	14.1 ± 0.2	48.1 ± 0.6 [2,b]	87.9 ± 0.6 [2,b]	71.5 ± 1.05 [1,b]	140.1 ± 2.0 [1,b]	64.7 ± 1.1 [b]	53.1 ± 0.5 [2,b]
**H^- 250**	22.7 ± 3.1	13.5 ± 3.4	3.9 ± 0.07 [2,a]	13.9 ± 0.3	48.6 ± 0.7 [2,b]	89.1 ± 0.7 [2,b]	62.8 ± 1.3 [2,a]	122.1 ± 2.5 [2,a]	57.6 ± 1.3 [a]	53.1 ± 0.6 [2,b]
**H^- 500**	27.6 ± 3.8	18.5 ± 4.2	4.2 ± 0.07 [2,b]	13.9 ± 0.3	45.7 ± 0.8 [2,b]	87.9 ± 0.7 [2,b]	69.4 ± 1.4 [1,b]	140.0 ± 2.6 [1,b]	62.4 ± 1.4 [b]	50.8 ± 0.7 [2,a]
**C**	24.5 ± 3.6	22.6 ± 3.9	4.6 ± 0.07 [1,b]	13.9 ± 0.3	45.4 ± 0.7 [2,b]	86.2 ± 0.7 [2,a]	71.0 ± 1.3 [2,b]	141.1 ± 2.4 [1,b]	63.5 ± 1.3 [b]	51.2 ± 0.6 [2,a]
**H^+ 125**	21.7 ± 4.4	21.6 ± 4.8	4.4 ± 0.2 [1,b]	15.3 ± 0.8	57.0 ± 1.9 [1,b]	93.6 ± 1.8 [1,b]	73.1 ± 3.4 [1,b]	125.4 ± 6.4 [2,a]	68.9 ± 3.5 [b]	60.1 ± 1.6 [1,b]
**H^+ 250**	28.6 ± 4.4	14.5 ± 4.8	4.2 ± 0.09 [2,b]	13.1 ± 0.4	42.9 ± 1.0 [2,a]	84.9 ± 0.9 [2,a]	64.1 ± 1.7 [2,a]	131.2 ± 3.3 [2,b]	56.7 ± 1.8 [a]	49.3 ± 0.8 [2,a]
**H^+ 500**	25.3 ± 6.2	14.4 ± 6.8	4.1 ± 0.1 [2,a]	14.5 ± 0.6	49.8 ± 1.6 [2,b]	90.0 ± 1.5 [1,b]	66.9 ± 2.8 [1,a]	130.3 ± 5.2 [1,a]	60.5 ± 2.8 [a]	54.1 ± 1.3 [2,b]

C control group; H^- heparin-non-binding fraction in concentrations 125 µg/mL, 250 µg/mL, 500 µg/mL; H^+ heparin binding fraction in concentrations 125 µg/mL, 250 µg/mL, 500 µg/mL; TMOT total motility, PMOT progressive motility

1,2 indexes indicate significant differences ($p < 0.05$) from the highest value (grey colour) of kinematic parameter in all groups

a,b indexes indicate significant differences ($p < 0.05$) from the lowest value (blue colour) of kinematic parameter in all groups

Table S2. Effect of SP protein fractions in three concentrations on distribution of motile sperm to subpopulations (slow, medium, fast).

**protein fraction**	concentration	subpopulations		
		slow	medium	fast
H⁻	125 µg/mL	34.1 %	35.7 %	30.2 %
	250 µg/mL	40.3 % +++	39.4 %	20.3 % – – –
	500 µg/mL	27.8 % – –	44.2% +++	28.0 %
C	0	30.9 %	34.9 % –	34.3 % +++
H⁺	125 µg/mL	29.1 %	37.7 %	33.2%
	250 µg/mL	36.7 %	35.6 %	27.7%
	500 µg/mL	43.2 % ++	30.9 %	25.9%
C	0	30.9 %	34.9 %	34.3%

C control group, H^- heparin-non-binding fraction in concentrations 125 µg/mL, 250 µg/mL, 500 µg/mL,
H^+ heparin binding fraction in concentrations 125 µg/mL, 250 µg/mL, 500 µg/mL
Grey and blue colour indicate the highest and lowest value of kinematic parameter in particular subpopulation, respectively
Number of + and – indicate strength of significance

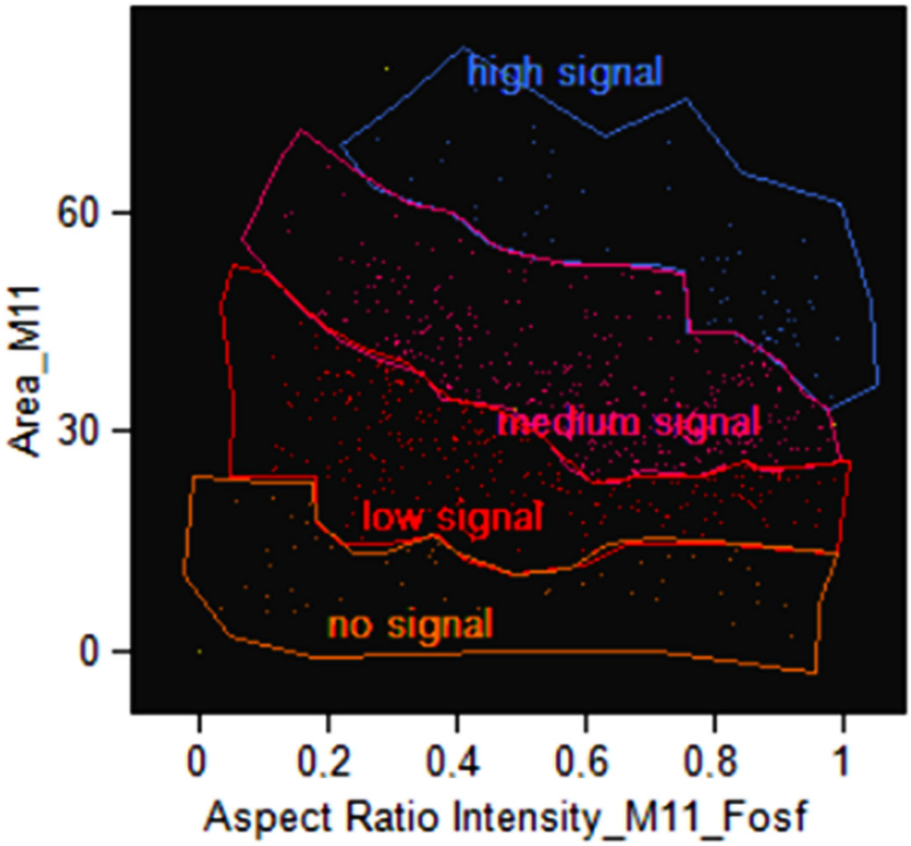

Figure S1. IDEAS spot-dot graph to evaluate image-based flow cytometry measurement of sperm protein phosphorylation using anti-phosphotyrosine antibody (4G10).
